# Lipid and metabolic profiles in female dogs with mammary carcinoma receiving dietary fish oil supplementation

**DOI:** 10.1186/s12917-019-2151-y

**Published:** 2019-11-08

**Authors:** Keidylania Costa-Santos, Karine Damasceno, Ricardo Dias Portela, Ferlando Lima Santos, Genira Carneiro Araújo, Emanoel Ferreira Martins-Filho, Laís Pereira Silva, Thiago Doria Barral, Stefanie Alvarenga Santos, Alessandra Estrela-Lima

**Affiliations:** 1 Escola de Medicina Veterinária e Zootecnia, Universidade Federal da Bahia, Salvador, Bahia 40170-110 Brazil; 2 Instituto Federal de Educação, Ciência e Tecnologia do Sertão Pernambucano, Santa Maria da Boa Vista, Pernambuco 56380-000 Brazil; 3 Instituto Gonçalo Moniz, Fundação Oswaldo Cruz, Salvador, Bahia Brazil; 4 Instituto de Ciências da Saúde, Universidade Federal da Bahia, Salvador, Bahia 40110-100 Brazil; 5 Universidade Federal do Recôncavo da Bahia, Santo Antônio de Jesus, Bahia 44570-000 Brazil; 6 Universidade do Estado da Bahia, Salvador, Bahia 41195-001 Brazil

**Keywords:** Breast cancer, Obesity, Lipids

## Abstract

**Background:**

Dyslipidemias induce angiogenesis and accelerate the development and in vitro growth of breast tumors. The aim of this study was to assess the lipid and metabolic profile of female dogs with mammary carcinomas and their correlations with body condition score and degree of tumor malignancy, as well as to study the effect of dietary fish oil supplementation on these animals.

**Results:**

Overweight or obese dogs had more aggressive carcinomas and higher triglyceride (*p* = 0.0363), VLDL (*p* = 0.0181), albumin (*p* = 0.0188), globulin (*p* = 0.0145) and lactate (*p* = 0.0255) concentrations. There was no change in the lipid profile after supplementation with fish oil at any concentration. However, in relation to the metabolic profile, glucose (*p* = 0.0067), total protein (*p* = 0.0002) and globulin (*p* = 0.0002) concentrations were increased when 90% omega-3 fish oil was used as a dietary supplement.

**Conclusion:**

Obese dogs showed altered lipid and metabolic profiles and more aggressive tumors, suggesting an important relationship between dyslipidemia and tumor aggressiveness. Supplementation with fish oil, rich in omega-3 fatty acids, may alter metabolic parameters in cancer patients.

## Background

Recent studies have demonstrated the influence of obesity on the development of different neoplasms in humans, such as neoplasms of the digestive tract including the esophagus, stomach, liver, gallbladder, pancreas, colon and rectum, as well as hormone-dependent neoplasms, such as breast, endometrial, uterine cervical and ovarian tumors [1]. Among the factors that link obesity to carcinogenesis are the activation of the insulin/IGF-1 pathway, increased concentrations of pro-inflammatory cytokines (TNF-α, IL-1 and IL-6) and their influence on adipocytokines [2], and dyslipidemia, which is often associated only to obesity [3]. However, changes in the lipid profile have been highlighted as a risk factor for neoplasm development, independent of overweight [4].

Literature data shows that dyslipidemias are potential factors associated with accelerated tumor formation [5]. Studies with rodents have demonstrated that hypercholesterolemia induces the formation of more aggressive tumors with rapid evolution and a greater predisposition to develop metastasis [6, 7]. In humans, this phenomenon has already been observed in prostate, colon and kidney cancer [2, 8]. In addition, triglycerides [9], low-density lipoprotein (LDL) [10] and very low-density lipoprotein (VLDL) cholesterol [11] are significantly elevated in women with breast cancer. In 2017, Lu [12] found that LDL and VLDL-C subfractions increased tumorigenesis of MCF7 and MDA-MB-231 neoplastic cells in vitro*.* This suggests that the control of hyperlipidemias is fundamental in cancer prevention.

One way to control hyperlipidemia in humans is supplementation with polyunsaturated fatty acids [13], such as fish oil, which is rich in polyunsaturated fatty acids of the omega-3 type. There are different forms of omega-3, main dietary long-chain omega-3 polyunsaturated fatty acids (LC-omega-3 PUFAs) eicosapentaenoic acid (EPA, 20:5ω-3) and docosahexaenoic acid (DHA, 22:6ω-3) [14] which have been widely studied because of their benefits [15]. This supplementation, according to in vitro research, was able to inhibit carcinogenesis by retarding the growth of breast, uterine, bladder, neuroblastoma, and glioblastoma tumor cells, and it can increase the efficacy of chemotherapeutic drugs [16]. Long-chain omega-3 polyunsaturated fatty acids can decrease mammary tumor growth and enhance survival in mice [17] and its synergy with the gut microbiota helps the immune system to overcome the immunosuppressive tumor microenvironment [18]. However, it has already been described that there is a positive association between long chain omega-3 fatty acids ingestion and cancer development [14]; however, some authors stated that this association is restricted to specific genetic variations [19] or even to toxic/carcinogenic compounds often accumulated along the food chain in the main long chain omega-3 dietary sources [20].

Despite the evidence that demonstrates the relevance of dyslipidemia to the occurrence of mammary neoplasms, research relating obesity and alterations in the lipid profile in the development of canine mammary tumors are scarce. For these reasons, our objectives were to identify the lipid and metabolic profiles of female dogs with mammary carcinomas, to verify the possible correlations of these profiles with body condition score and degree of tumor malignancy and to study the effect of fish oil supplementation as a nutraceutical therapy for female dogs with mammary carcinoma.

## Results

### Clinical, reproductive and pathological factors

Poodles were the most common breed among the subjects (23.81%, 10/42), followed by mixed breed dogs (19.05%, 8/42) and Miniature Pinschers (14.29%, 6/42). The other breeds represented 42.86% of the sample (18/42). Regarding reproductive characteristics, 9 of the 42 female dogs (21.43%) had been spayed before the presence of the nodule, and the other 33 (78.57%) were not spayed. It was also noted that five female dogs (12.19%) had been medicated with injectable contraceptives and 18 female dogs (42.86%) had had pseudopregnancies at some time in their lives. Twenty-eight female dogs were diagnosed with CMT, and 14 female dogs with other carcinomas (MC). The clinical-pathological findings are summarized in Table 1.

**Table 1:** Distribution of animals according to groups and clinical-pathological findings

Group	Histopathological Diagnosis	Grade	Staging (TNM)	
MC	Tubular Carcinoma	Grade I (*n* = 03)	Stage I	(*n* = 02)
(*n* = 14)	(*n* = 4)		Stage II	(*n* = 01)
		Grade II (*n* = 01)	Stage III	(*n* = 01)
	Papillary Carcinoma	Grade I (*n* = 03)	Stage I	(*n* = 03)
	(*n* = 6)		Stage II	(*n* = 02)
		Grade II (*n* = 03)	Stage III	(*n* = 01)
	Solid Carcinoma	Grade I (*n* = 02)	Stage I	(*n* = 02)
	(*n* = 4)	Grade II (*n* = 02)	Stage I	(*n* = 01)
			Stage II	(*n* = 01)
CMT		Grade I (*n* = 25)	Stage I	(*n* = 10)
(*n* = 28)			Stage II	(*n* = 08)
			Stage III	(*n* = 07)
		Grade II (*n* = 03)	Stage III	(*n* = 02)
			Stage IV	(*n* = 01)

### Body condition scores of female dogs with mammary cancer

It was verified that 11 female dogs (26.20%) were below the standard BCS, 10 (23.80%) were within the standard range and 21 (50%) were above the reference range. When correlating reproductive status with body status, we observed a significant increase in the BCS (*t* = 2.18, *p* = 0.035) of spayed animals (16.32 ± 4.79) compared to unspayed animals (13.49 ± 3.37).

### Lipid profile of female dogs diagnosed with CMT and MC

Triglycerides and VLDL-C showed differences (*p* < 0.05) in the comparison between histological groups, with greater values in the group with a higher degree of malignancy. When evaluating the triglyceride concentration, we observed a difference in the mean concentration in relation to the histological type (*p* = 0.0363). Females with CMT (pre = 54.89 ± 5.38, post = 62.31 ± 11.03) had lower mean values than females with MC (pre = 91.23 ± 20.42, post = 71.96 ± 9.803). However, no difference was observed between TG (*p* = 0.6841) and VLDL-C (*p* = 0.9196) concentrations before and after surgery (*p >* 0.6841; Table 2).

**Table 2:** Maximum likelihood means for variables referring to serum component concentrations evaluated in female dogs considering cancer type, presence/absence of tumors and their interaction, with standard error of the mean (SEM) and *p*-value for the factors/interactions. Values with probability less than 5% (*p* < 0.05) were considered statistically significant

		Type		Mastectomy			*p*-value	
	CMT	MC	PRE	POST	SEM	Type	Mastec	Type*Mastec
Cholesterol	5.29	5.37	5.35	5.30	0.058	0.2003	0.4178	0.5766
Triglycerides	3.88	4.19	4.07	4.01	0.144	0.0363	0.6841	0.6418
HDL	3.49	3.71	3.39	3.81	0.251	0.3968	0.0995	0.7918
VLDL-C	2.27	2.66	2.46	2.48	0.159	0.0181	0.9196	0.9626
LDL	4.93	4.65	4.92	4.67	0.145	0.0603	0.1010	0.7159
Protein	1.97	1.82	1.94	1.84	0.077	0.0517	0.1987	0.9483
Albumin	0.93	1.17	0.99	1.11	0.101	0.0188	0.2800	0.1207
Glucose	4.43	4.39	4.49	4.33	0.092	0.6901	0.0778	0.5528
Calcium	2.21	2.27	2.21	2.27	0.057	0.2728	0.3307	0.1106
Lactate	3.77	4.18	3.88	4.07	0.179	0.0255	0.3068	0.5437

According to the maximum likelihood means, which evaluate the influence of one parameter on the other, that is, the relationship between tumor type and tumor presence, there was no significant change in VLDL-C concentration (*p* = 0.9626; Table 2) nor for both CMT (*p* = 0.4938) and MC (*p* = 0.3745) type. However, it was found that the MC group with the highest malignancy had an elevated VLDL-C concentration (*p* = 0.0181) (pre CMT = 10.98 ± 1.075, post = 12.46 ± 2.206, pre MC = 18.25 ± 4.084, post = 14.39 ± 1.960). No influence of the interaction between histological type and tumor presence on the serum total cholesterol, HDL, or LDL concentrations was observed (*p* = 0.5766; *p* = 0.7918; *p* = 0.7159, respectively).

**Fig. 1:**
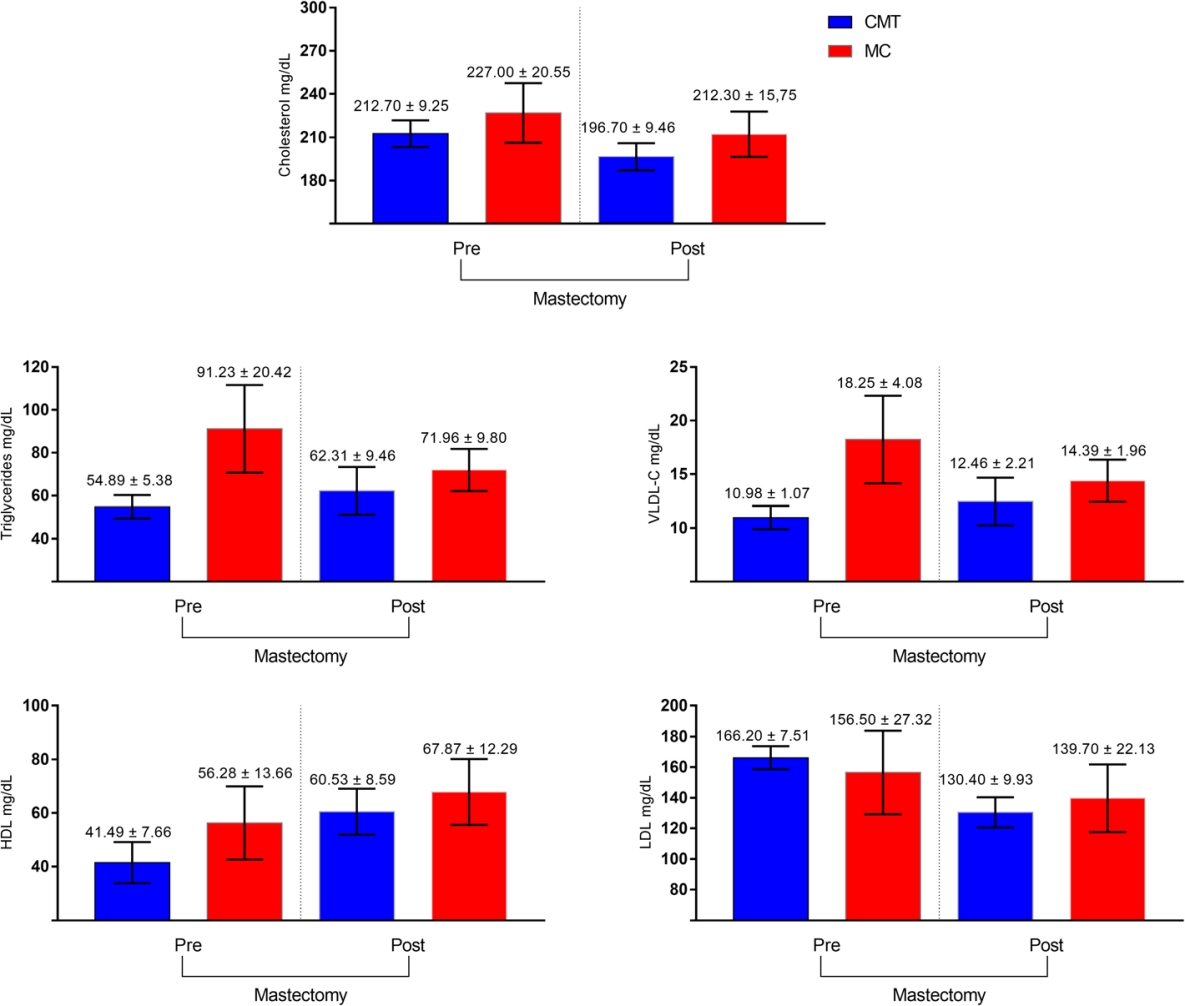
Lipid profile of female dogs with mammary carcinoma before and after mastectomy (*p < 0.05). Blood collection 2 days before and 15 days after the mastectomy. CMT, carcinoma in mixed tumors; MC, other carcinomas; LDL, low-density lipoprotein; HDL, *high-density lipoprotein;* VLDL, very low-density lipoprotein

The presence or absence of a tumor did not significantly alter the VLDL-C concentration (*p* = 0.9196) (Fig. 1). The concentrations of total cholesterol, HDL cholesterol and LDL did not vary between histological groups (*p* = 0.2003, *p* = 0.3968, *p* = 0.0603, respectively) or after tumor removal (*p* = 0.4178, *p* = 0.0995; *p* = 0.1010). Similarly, there was no significant interaction between histological type and tumor presence (*p* = 0.5766; *p* = 0.7918; *p* = 0.7159).

### Metabolic profile of female dogs diagnosed with CMT and MC

Total protein, glucose and calcium did not show alterations according to different histological types (CMT and MC) (*p* = 0.0517; *p* = 0.6901; *p* = 0.2728, respectively), excision of the tumor (*p* = 0.1987; *p* = 0.0778; *p* = 0.3307), or the interaction of these variables (*p* = 0.9483; *p* = 0.5528; *p* = 0.1106).

**Fig. 2:**
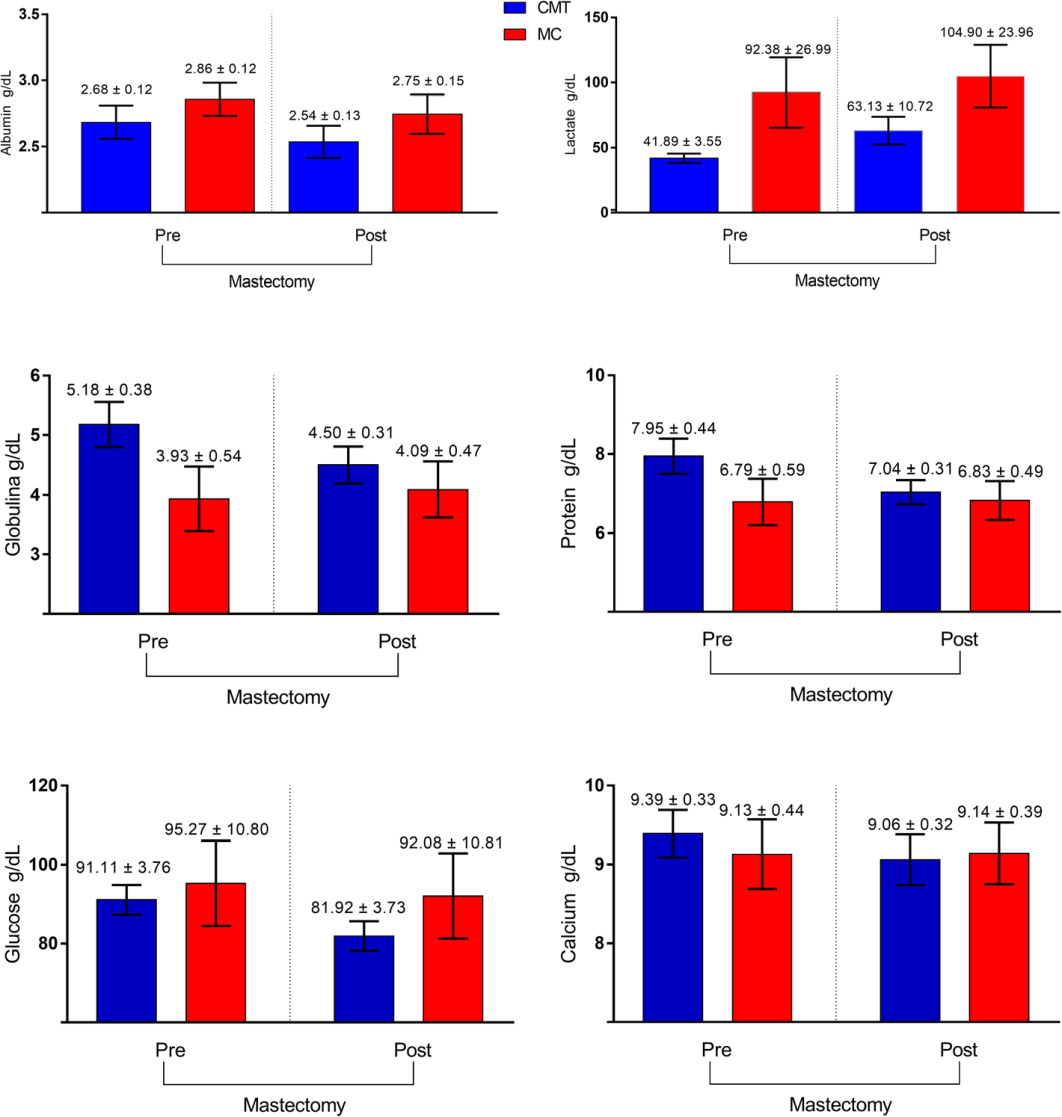
Total protein, albumin, glucose, calcium, lactate and globulin concentrations in female dogs with mammary carcinoma before and after mastectomy (*p < 0.05). Blood collection 2 days before and 15 days after the mastectomy. CMT, carcinoma in mixed tumors; MC, other carcinomas

Lactate showed significant differences regarding tumor type (*p* = 0.0255). But there were no significant differences for tumor presence (*p* = 0.2800 and *p* = 0.3068). In the group of CMT dogs after total unilateral mastectomy, albumin (CMT pre = 2.685 ± 0.13, post = 2.53 ± 0.12, *p* = 0,1376) was slightly below the reference value (2.6–3.3 g/dL), and lactate (CMT pre = 41,89 ± 3.55, post = 63.13 ± 10.72, *p* = 0,1039) was below the reference range before mastectomy (45–233 U/L). The mean concentration of globulin (CMT pre = 5.17, post = 4.5) was elevated in the CMT group, considering 2.7–4.4 g/dL the reference range (Fig. 2).

### Lipid and metabolic profiles after supplementation with omega-3

**Fig. 3:**
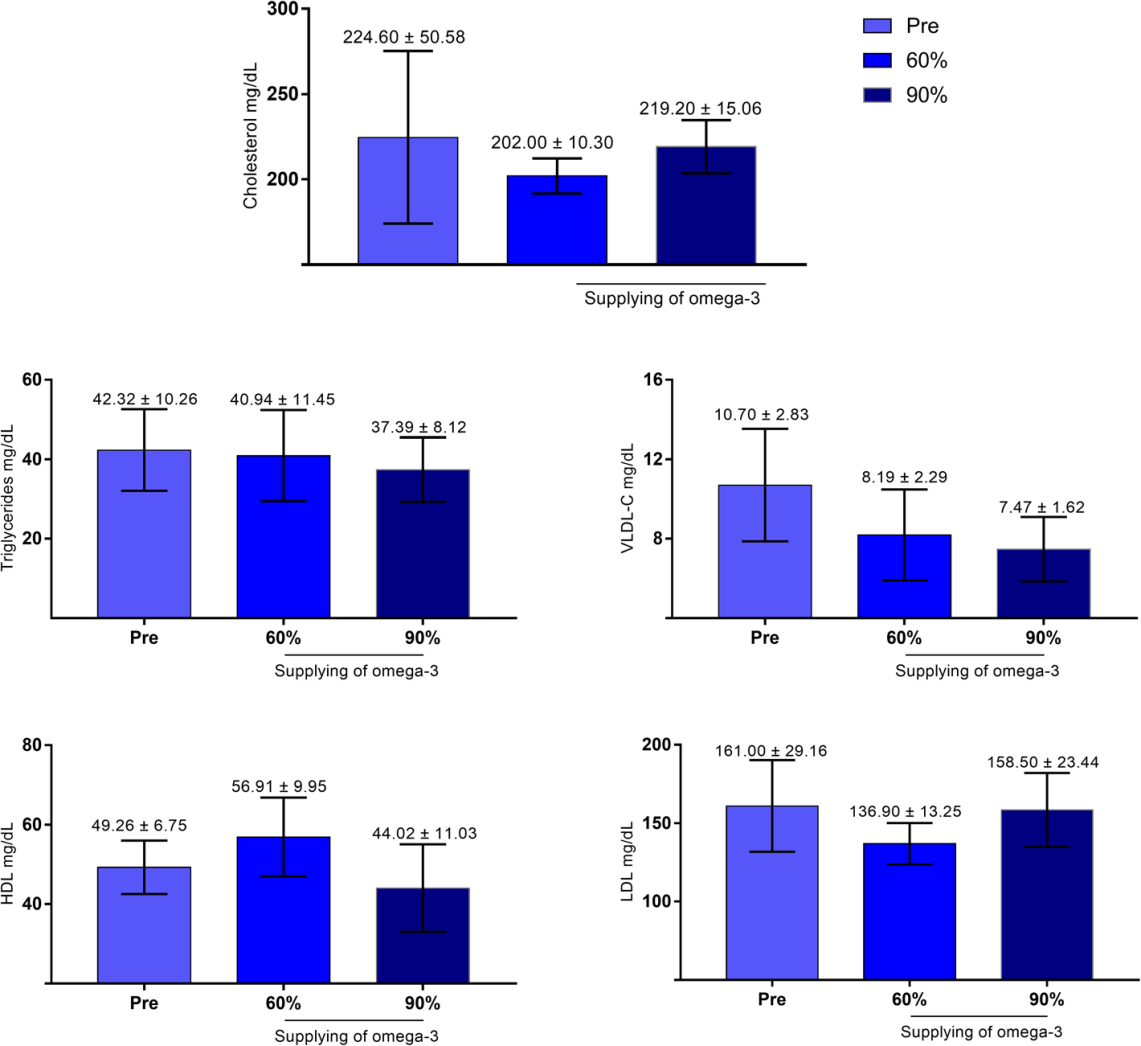
Lipid profile of female dogs with mammary carcinoma before, during and after receiving supplementation with 60 and 90% omega-3 (*p < 0.05). Blood collection 15 days after the surgical procedure, at the beginning of the supplementation, 30 and 60 days after the start of supplementation with fish oil (omega-3)

**Fig. 4:**
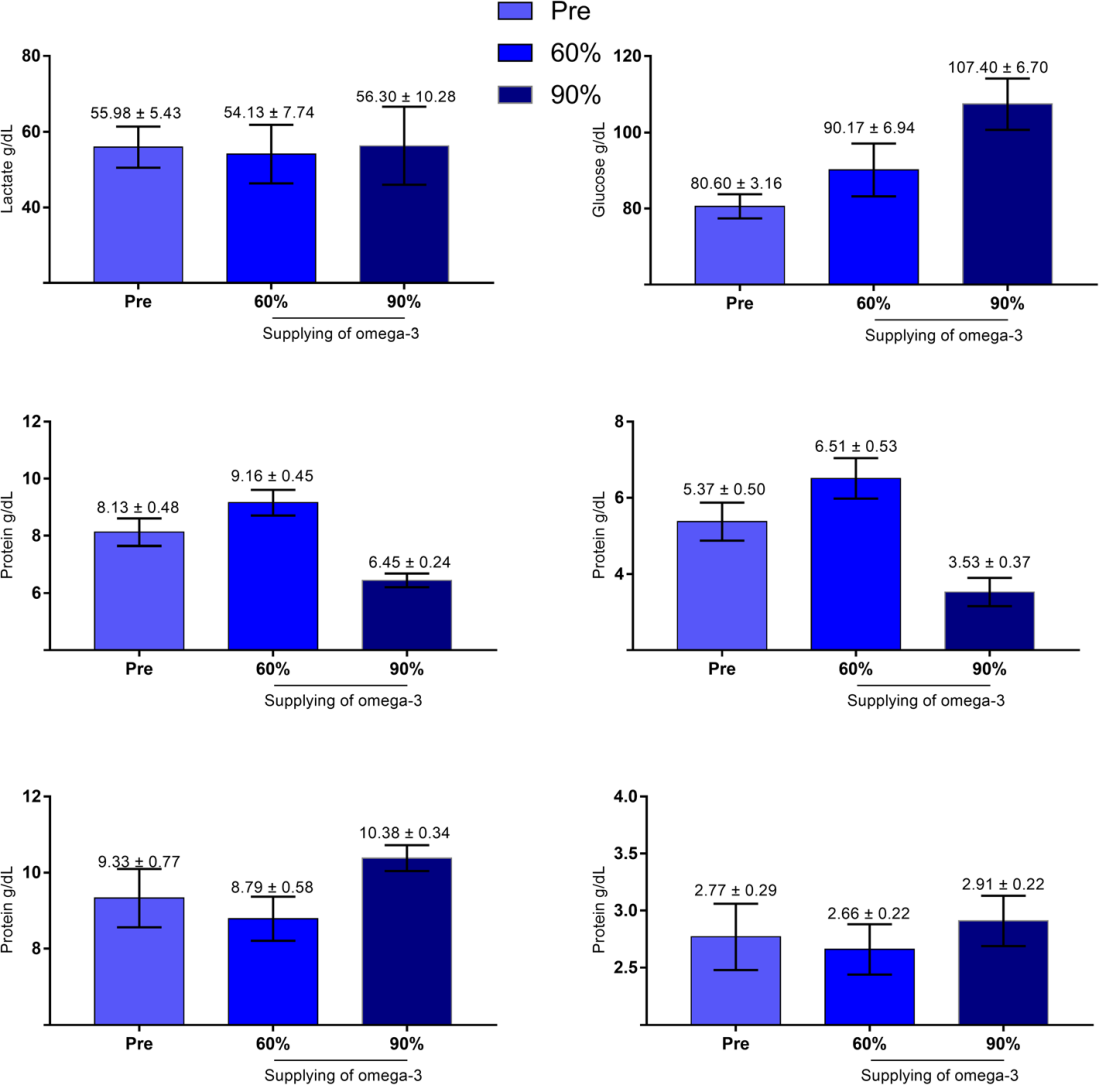
Metabolic profile of female dogs with mammary carcinoma before, during and after receiving supplementation with 60 and 90% omega-3 (*p < 0.05). Blood collection 15 days after the surgical procedure, at the beginning of the supplementation, 30 and 60 days after the start of supplementation with fish oil (omega-3)

There was no change in the lipid profile after supplementation with fish oil at any concentration (Fig. 3). However, when the influence of different concentrations of omega-3 on the metabolic profile was evaluated by means of maximum likelihood, changes in glucose, protein and globulin were observed when fish oil with 90% omega-3 was administered (Table 3). In female dogs supplemented with 90% omega-3, higher glycemic indexes (pre = 80.60; O-90% 107.4; *p* = 0.0067) and lower serum globulin (pre = 5.366; O-90% 3.52; *p* = 0.0002) and serum protein levels (pre = 8.133; O-90% = 6.44; *p* = 0.0002) were observed after supplementation than before surgery (Fig. 4).

**Table 3:** Maximum likelihood means for variables at different concentrations of omega-3, with the standard error of the mean (SEM) and *p*-value. Values with probability less than 5% (*p* < 0.05) were considered statistically significant

					*p*-value	
Omega-3	Pre	60	90	SEM	Pre × 60	Pre × 90
Triglycerides	35.404	34.800	34.573	0.279	0.8817	0.8772
Cholesterol	53.950	53.008	53.757	0.067	0.3348	0.7390
HDL	38.330	39.319	38.341	0.189	0.7080	0.8407
LDL	49.268	48.881	50.070	0.191	0.8843	0.6829
VLDL-C	21.100	18.704	18.476	0.283	0.5574	0.6859
Glucose	43.852	44.847	46.646	0.061	0.2658	0.0067
Albumin	0.9775	0.9543	10.539	0.098	0.8699	0.4750
Protein	20.851	22.081	18.592	0.050	0.0989	0.0002
Calcium	22.121	21.605	23.364	0.065	0.5811	0.0753
Lactate	39.954	39.262	39.324	0.147	0.7433	0.8764
Globulin	20.851	22.081	18.592	0.050	0.0989	0.0002

### Survival

**Fig. 5:**
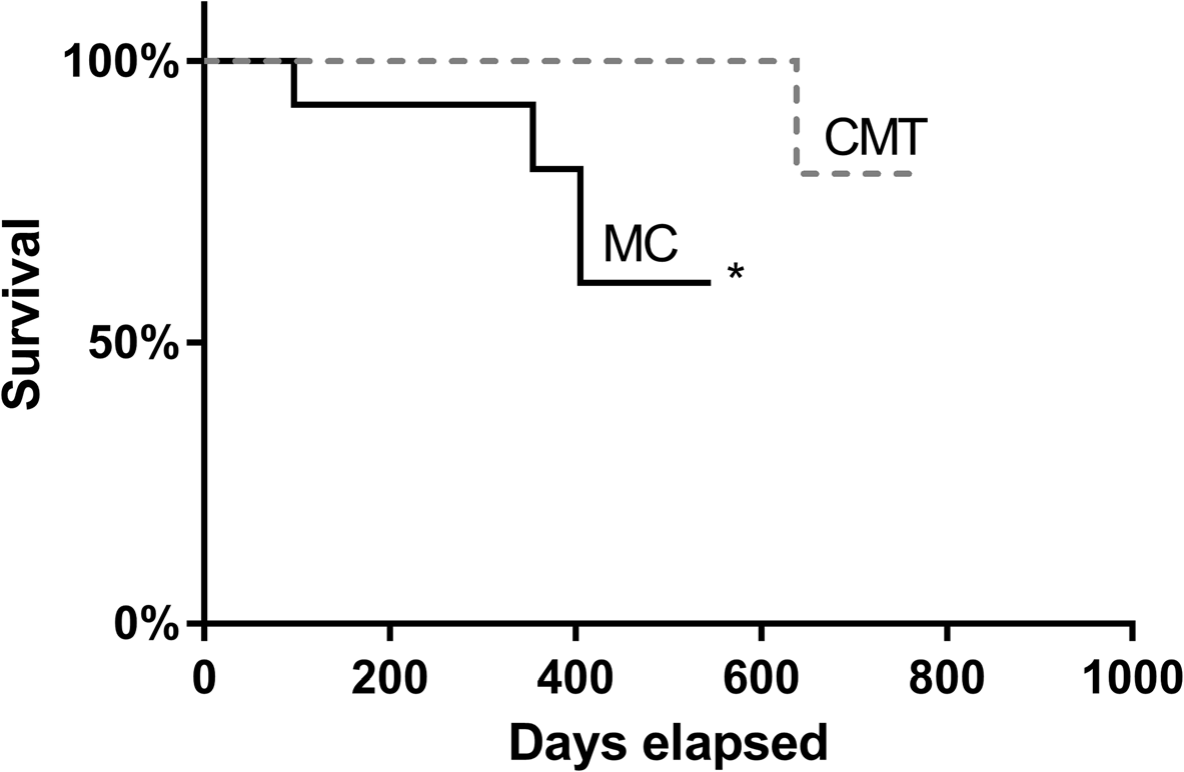
Survival curves of the CMT and MC groups. Significant differences when *p* < 0.05, highlighted by *

The minimum survival time was 97 days, attributed to an animal in the MC group, which died due to pulmonary metastasis after the total unilateral mastectomy, and the maximum time was 638 days after mastectomy, attributed to an animal in the CMT group. Based on the evaluated survival curve stratified by group, significant values were observed (*p* = 0.0076) for the CMT group relative to the MC group (Fig. 5). The survival of the female dogs that ingested fish oil (331.0 ± 20.91) was not significantly different from those that did not use the supplements (289.6 ± 21.30) during the time period studied.

## Discussion

This is the first research work that investigates the influence of omega-3 intake in the survival and clinical biochemistry profile of female dogs undergoing mammary cancer treatment. The results of this study showed a change in the lipid and metabolic profiles of obese dogs that also have especially aggressive carcinomas, thus suggesting a possible influence of these factors on tumor malignancy. The influence of omega-3 fatty acids on the metabolic profile was also demonstrated. It should be emphasized that two different histological groups of tumors were herein studied, the less aggressive CMT, and the MC with a high malignity potential. Regarding reproductive characteristics, the higher proportion of unspayed than spayed females in the sample with neoplasia was already expected, as sex hormones are recognized to participate in the etiology of mammary cancer [21].

The main method for prevention of mammary neoplasms in dogs is early ovariohysterectomy (OH), which minimizes the risk of mammary nodules [22]. Castration as a protective factor against mammary cancer is unquestionable. However, early castration is known to delay the closure of long bone fissures, predispose the animal to recurrent vulvar fold dermatitis, and trigger a strong tendency towards body weight gain, which can culminate in obesity [23]. A reduced frequency of obesity was observed in unspayed female dogs compared to spayed ones (80%), corroborating previously observed results [24]. Ovariohysterectomy can result in significant weight gain after surgery in bitches fed ad libitum and the weight gain is exclusively a fat gain [25, 26]. Spayed females present a higher food ingestion, indiscriminate appetite and a twofold higher risk to develop obesity [27]. The spay process does not directly cause a metabolic problem but induces changes in appetite that contribute to obesity development. Indeed, obesity was already associated with several neoplasms [28].

Obese postmenopausal women face a twofold risk of developing breast cancer, and these tumors are mostly hormone-dependent [29]. The pathways that link breast cancer and obesity in women have not yet been completely elucidated. However, some mechanisms have been suggested: the deregulation of insulin signaling can lead to an imbalance in cell differentiation, proliferation, apoptosis rates as well as altered adipokine expression [30]. An increased production of pro-inflammatory markers can maintain a state of chronic inflammation [31]. Alterations in the metabolism of sex hormones in obese women might reduce the concentration of androgens [32] and compromise the production of estrogen (a hormone implicated in mammary carcinogenesis), resulting in the aromatization of androstenedione in the adipose tissue of post menopause women [29, 33, 34]. The latter mechanism might also contribute significantly to the hormonal support of the tumor in spayed and obese dogs (unpublished data). Thus, in view of the important relationship among obesity, carcinogenesis and tumor aggressiveness [35], it is important to evaluate the possibility of performing castration between the first and second estrous cycles, guaranteeing full bone, muscle, immunological and endocrine development as well as contributing to the effort to prevent body weight gain.

Clinical stage I, consisting of tumors up to three centimeters without metastasis to regional lymph nodes (T1), was the predominant stage and predicted a relatively good prognosis. These results contrast with another study [36], which demonstrated a predominance of mammary tumors larger than five centimeters (T3) with regional (T4) or distant (T5) metastasis. This change in patient profile suggests that the owners of the dogs in the present study took greater care with their companion animals and sought veterinary medical treatment early, preventing the advancement of neoplasia. Early diagnosis of mammary cancer can reduce mortality and results in less effective chemotherapeutic intervention [36].

Regarding histological types, we observed a predominance of mixed tumor carcinoma (CMT) of grade I. This tumor derives from the malignant transformation of the epithelial component of a benign mixed tumor and is considered, in principle, a tumor with a relatively good prognosis. However, its histological grade should always be considered because of the direct correlation with its malignancy potential. The results of this study were similar to those found in other studies [37–39].

In the present study, a high concentration of VLDL-C was observed in the more aggressive group of carcinomas (MC), which could suggest the participation of this lipoprotein in tumor aggressiveness. To investigate the effects of VLDL-C on viability, angiogenesis, cell motility and multiplication stimulus (metastasis) in MCF-7 and MDA-MB-231 mammary lineage tumor cells, Lu [12] submitted the cells to in vitro treatment with LDL, VLDL-C and HDL and found that VLDL-C increased the viability of human umbilical vein endothelial cells (HUVECs) and the formation of endothelial microvessels. Compared to the other lipoproteins, VLDL-C increased the phosphorylation of AKT Ser473, a survival signal that ameliorates migration and invasion potential, and furthermore increased the viability of endothelial cells by increasing the concentration of amphiregulin (AREG) [12]. AREG belongs to the EGFR family and participates in different aspects of tumorigenesis. Considering the high concentration of VLDL observed in the MC group, internal tumor vascular density should be investigated in future studies.

The concentration of lactate was significantly different between histological groups. Aerobic oxidation and glycolysis are two forms of glucose metabolism that respond to distinct conditions of oxygen demand. The glycolysis rate is abnormally high in tumor cells, regardless of the availability of oxygen; this phenomenon is called the Warburg effect [40]. Because of the high glycolysis rate, the production of lactate is increased, creating an acidic microenvironment in the tissues. Normal cells become necrotic due to changes in the transmembrane gradient of H^+^ and the degradation of the extracellular matrix in an acid microenvironment [41]. However, tumor cells resist acidosis, surviving at low pH. In addition, acidosis can inhibit the immune response to tumor antigens, promoting the proliferation and invasion of tumor cells [42]. The lactate output induces the proliferation of malignant oxygenated cells, promotes angiogenesis, and inhibits the innate and adaptive immune responses. For this reason, the elevation of serum basal lactate is associated with reduced survival [43]. These events can explain the higher concentration of lactate in the group with greater malignancy, in addition to the weight loss in some obese female dogs with mammary carcinoma. Despite the significant difference between groups and the concentration of lactate being higher in the MC group than in the CMT group, the average is still within the reference range.

The elevated concentration of globulin in female dogs with CMT is probably related to the increase in the concentration of hormone binding globulins. This type of globulin specifically binds to testosterone and estradiol, thereby functionally regulating the concentration of free steroidal hormones [44]. The main results include increased glucose and decreased globulins during omega-3 treatment and absence of altered lipid profile during the period of omega-3 supplementation.

By examining the effects of omega-3 fatty acid supplementation on HDL cholesterol, it can be observed an increase, without a significant difference, in female dogs that received 60% omega-3 fish oil. This phenomenon was not observed during the period of the higher concentration of omega-3 supplementation. This effect of n-3 fatty acids, characterized by a sharp reduction in the concentration of HDL, is common in animals but is not usually observed in humans. These differences between animals and humans arise not only from the differences underlying lipoprotein metabolism but also from the differences in experimental models, in which larger amounts of n-3 fatty acids are supplied to animals than to humans [45], and our dosing was considered to be a very modest anti-inflammatory dose when compared to other studies in rodents.

The VLDL-C values decreased as the concentration of omega-3 increased. Ma [46] observed that omega-3 could inhibit angiogenesis through the intermediate metabolite PGE3 by inhibiting the proliferation and migration of HUVECs. Considering the possible involvement of omega-3 fatty acids in the process of angiogenesis, the decrease in the concentration of this lipoprotein may be an effective way to reduce the risk of neoplasms.

Sex hormones have an important role in the etiopathogenesis of mammary cancer, and sex hormone binding globulin modulates the functions of testosterone and estradiol, changing their bioavailability in the organism. Therefore, we recommend studying whether the increase in globulin with omega-3 supplementation is related to increased hormone binding globulin and whether it can function as a positive marker, as it provides an active mechanism for omega-3 use in the prevention of hormone-dependent neoplasms.

The utilization of omega-3 led to increased blood glucose in female dogs, suggesting that glycemic metabolism may change with high concentrations of omega-3. A trend has been observed in which fish oil, particularly the omega-3 fatty acid EPA in capsule form, raises the fasting glucose levels of hyperlipemic men after ingestion [47]. However, low doses of this type of omega-3 (900 and 1800 mg/d) were not able to change the concentration of glucose or glycated hemoglobin in humans [48]. The mechanism by which fatty acids affect blood glucose includes an increase in the liver production of glucose, which may be related to the increased flow of gluconeogenesis precursors in the in liver [47, 49].

Total protein levels in serum increased significantly with 90% omega-3 supplementation. Because of the lack of significant change in the albumin concentration, the increase in total protein might be related to the increase in globulin because the total serum protein content comprises albumin and globulins without fibrinogen [48].

Finally, when analyzing the survival time and survival rate of female dogs with mammary carcinoma, we were able to observe that female dogs with CMT had a longer survival time than those in the MC group. This observation is expected because most of the tumors evaluated in this study received a histopathological diagnosis of CMT grade I, that is, a neoplasm of lower malignancy than the other grades or histological types [38]. In the female dogs that ingested omega-3 there was no direct significant effect on survival time. Further long-term studies with omega-3 are needed to evaluate a possible influence on survival time. It should be noted that the omega-3 treatment was made only for 2 months; thus, it is possible that the action of the treatment would be different or potentiated over a prolonged period of ingestion. Another point to be considered is the dose used, which may be considered low when compared to a previous study [50], but was in the range of doses recommended by some manufacturers. In addition, the relationship between different types of omega-3 fatty acids (omega-3 and omega-6) is reported to be more important than just the concentrations [51, 52].

## Conclusions

Obese female dogs have more aggressive tumors and mild alterations of components of lipid and metabolic profiles, suggesting a relationship between tumor aggressiveness and VLDL-C, triglyceride and lactate concentrations. Thus, overweight/obesity may be a risk factor for the incidence and progression of mammary nodules, mostly in spayed female dogs. Supplementation for longer than 90 days with fish oil, rich in omega-3 fatty acids, may help control dyslipidemia and obesity in dogs.

## Methods

### Animals

A total of 42 female dogs of different breeds, ranging in age from six to 18 years (9.9 ± 2.4 years, 95% CI (9.1–10.6)), diagnosed with mammary carcinoma, admitted and cared for at the Professor Renato Rodemburg de Medeiros Netto Veterinary Medicine Hospital from April 2015 to March 2017, were evaluated. The female dogs were divided according to cancer histological type: carcinoma in mixed tumors (CMT) or other carcinomas (MC). The animals eligible for the present study had not used antineoplastic drugs or fish oil supplements for at least thirty days before surgery and had no history of endocrinopathy. Genetic or idiopathic high concentrations of cholesterol and triglyceride have been reported to be familial in certain pure breeds, such as Miniature Schnauzers, Shetland Sheepdogs, Labrador Retrievers, Miniature Dachshunds and Shiba Inus. Additionally, the magnitude of the abnormal total cholesterol (T-Cho) and total triglyceride (T-TG) levels in these breeds tends to differ with dog age and sex [53, 54]. Thus, all animals evaluated were females; breeds predisposed to dyslipidemias were not included in the study and it was considered the equivalence between age frequencies in the animals of the two groups studied (CMT and MC).

Concerning the dog breeds, it was observed a higher frequency of poodles (28.57% - 12/42), followed by dogs with no defined race (23.80% - 10/42) and Miniature Pinschers (14.28% - 6/42). The other breeds corresponded to 42.85% (18/42). Regarding the reproductive characteristics, 11 (26.19%) bitches had been spayed before the presence of the nodule and 31 (73.80%) were not spayed. Five bitches (11.90%) had already received contraceptive treatment, and 18 (42.85%) bitches presented pseudo-cysts at some time in their lives. The identification of the phase of the estrous cycle was not determined. However, no bitch was in estrus at the time of surgery.

The research was approved by the Animal Ethics Committee of Federal University of Bahia under protocol number 05/2015. A written informed consent was obtained from all the legal owners. All animals included in this study were screened monthly by a staff of veterinarian oncologists, when clinical and laboratory tests and thoracic radiological examinations were performed. Owners were instructed to contact the veterinarians in case of any side effect presented by the animals. The staff were prepared to perform prompt evaluation and treatment of these animals in case of any pain, suffering or severe symptoms, and even to evaluate whether the animal should undergo a more specific treatment or even humanitarian euthanasia (with the owner’s written consent), at which point animals were excluded from the study. The euthanasia procedure consisted of a deep anesthesia induced by propofol (a doses four times higher than the one used to induce anesthesia), followed by an intravenous administration of KCl.

### Clinical evaluation

The female dogs were initially submitted to a systematic clinical examination, and complementary exams were performed to identify regional and distant metastases to establish a complete staging categorization. Stage was determined from information on the tumor size (T), the involvement of regional lymph nodes (N) and the presence or absence of distant metastases (M). In this way, stages II, III, IV and V were defined based on the TNM system [55].

The clinical anamnesis was carried out through a detailed evaluation of the physiological parameters, the prior evolution of the disease and the animals’ reproductive records along with biochemical and hematological analyses. The macroscopic evaluation included determination of the tumor features (size, presence of inflammatory reaction and/or ulceration) and location and analysis of regional lymph nodes by palpation and aspiration cytology. The confirmation of neoplastic involvement was determined by histopathological analysis. Imaging exams (thoracic x-ray from three angles: right-lateral, left-lateral, and ventral-dorsal) were performed for pulmonary metastasis screening, and total abdominal ultrasonography for abdominal metastasis evaluation was also performed.

### Determination of body condition score

Female dogs were classified as adequate weight or overweight according to their body condition scores (BCS), and the scores ranged from 1 to 9. Animals with a score of 1 to 3 were considered lean, scores of 4 and 5 were classified as adequate body condition scores, and animals with a score from 6 to 9 were considered overweight [56].

### Surgery

The surgical approach herein used was the total unilateral mastectomy and the gonadectomy in unspayed female dogs. The female dogs were submitted to food and water fasting for 12 and 2 h, respectively. The administration of pre-anesthetic medication (chlorpromazine hydrochloride 0.03 mg/kg) was conducted intramuscularly. Then, the cannulation of the cephalic vein was performed for the infusion of Ringer’s solution at a rate of 10 mL/kg/hour flow rate. Immediately thereafter, the dogs were administered with meloxicam (0.2 mg/kg) and cephalothin (30 mg/kg), both intravenously. Then, hair was clipped, and an extensive antisepsis of the thoracic region and the ventral abdominal region was made, and the animals were placed on the surgery table. Twenty minutes after the administration of meloxicam and cephalothin, propofol was administered (5 mg/Kg) and the orotracheal intubation of the animals was made, followed by the administration of isofluorane in a concentration sufficient to reach an surgical anesthesia stage [57]. All animals were subjected to total unilateral mastectomy with removal of the inguinal lymph nodes and gonadectomy in unspayed female dogs. The physiological parameters were monitored with the aid of multiparametric monitor. A single team performed the anesthetic and surgical procedures, with a mean surgical time of 50 min. Female dogs were monitored during the postoperative period, and compression bandages were used to protect the sutures and improve healing. On the fifteenth day, the sutures were removed, and peripheral blood was sampled after a 12-h fasting period to evaluate the postoperative lipid and metabolic profiles.

### Histopathological procedure

Immediately after surgery, the mammary chain with regional lymph nodes was sent to the Laboratory of Veterinary Pathology (LPV) of the Federal University of Bahia for macroscopic and microscopic characterization of the tumor. The removed mammary chain and lymph nodes were fixed in phosphate-buffered 10% neutral formalin and processed by the routine technique of paraffin embedding [58]. Histological sections of 4 μm thickness were stained with hematoxylin and eosin [59] for subsequent classification based on histopathological diagnosis according to the World Health Organization (WHO) criteria complemented by the criteria proposed by Cassali et al. [60] and Goldschmidt [61]. The histological grade of the tumor was described by the Nottingham system as modified by Elston and Ellis [62], which evaluates the percentage of tubule formation, nuclear pleomorphism and the mitotic index.

### Peripheral blood sample collection

For the evaluation of the lipid and metabolic profiles, blood was collected with sterile disposable 5 mL syringes by jugular venipuncture and transferred to 10 mL sterile glass tubes. Then, the blood was centrifuged at 3000 rpm for 10 min to obtain the serum, which was stored and kept at − 20 °C for further analyses. The entire blood sample obtaining process was made in a fast and gentle way to avoid stress, and the blood samples were immediately sent to the clinical biochemistry laboratory to be processed.

### Evaluation of serum lipid profile

Blood samples for lipid profiling were collected 2 days before the mastectomy, 15 days after the mastectomy, and 30 and 60 days after the start of supplementation with fish oil (omega-3). Commercial biochemical kits (Labtest®, Lagoa Santa, Minas Gerais, Brazil) were used to measure total cholesterol, triglycerides and HDL cholesterol. Each collection was performed after a 12-h fast. The levels of VLDL and LDL cholesterol were obtained by the Friedewald’s formula [63]. The reference values herein considered were 135–270 mg/dL for total cholesterol, 60.0–140.0 mg/dL for HDL cholesterol, 34.0–115.0 mg/dL for LDL cholesterol; up to 25.0 mg/dL for VLDL cholesterol and 20–112 mg/dL for triglycerides [64, 65].

### Metabolic profile evaluation

The metabolic profile consisted of the serum levels of glucose, calcium, albumin, lactate and total protein. The evaluation was performed by means of peripheral blood collection and use of commercial clinical biochemistry kits, such as glucose, calcium, albumin, lactate and total proteins (Labtest®). The globulin levels were calculated by the following formula: Total proteins - albumin = globulins. The reference values [64, 65] were 70–110 mg/dL for glucose, 5.4–10 g/dL for total protein, 2.6–3.3 g/dL for albumin, 2.7–4.4 g/dL for globulin and 45–233 U/L for lactate. Only samples without lipemia were used for analysis.

### Supplementation with fish oil (omega-3)

Fifteen days after the surgical procedure, 28 female dogs started to ingest the fish oil capsules. The animals were randomly selected and the participation was later confirmed based on the owner’s commitment to administer the omega-3 capsules as prescribed. Two products containing fish oil, from the same brand (Inovet, Rio de Janeiro, Brazil) were used. The first one (90% EPA-DHA 1.5:1) was used during the first month of evaluation, and each capsule of the product contained 450 mg of pure fish oil, 270 mg of EPA (eicosapentaenoic acid) and 180 mg of DHA (docosahexaenoic acid), and the dose was one capsule for each 10 kg of body weight per day. The second product (60% EPA-DHA 1.5:1) was given for the next 30 days, and each capsule contained 300 mg of pure fish oil, 180 mg of EPA and 120 mg of DHA, and the daily dose was also one capsule for each 10 kg of body weight. The other compound present in the capsules was vitamin E, in the respective concentrations of 2222 IU/kg and 1490 IU/kg for each product described above. The dogs ingested the capsules mixed to its chow and under the owner’s supervision to ensure its whole ingestion. Blood was collected at the beginning of the supplementation period and 30 and 60 days after the beginning of the treatment. Flasks containing fish oil were kept at room temperature and away from light. The supplemented female dogs presented the following histopathological diagnoses: CMT (*n* = 12) and MC (*n* = 16).

### Monitoring and survival

Radiological examinations were performed every 60 days, and laboratory tests (hemogram, urea, creatinine, alanine aminotransferase and alkaline phosphatase) and clinical examinations were performed monthly for a follow-up period of 12 months after surgery, in which data were collected regarding overall survival. The overall survival time was defined (in days) as the period between surgical excision of the primary tumor and the date of death from the disease. With written consent from the owners, the animals that died were submitted to necropsy with the objective to determine the *cause of death* and detecting possible metastasis.

### Statistical analysis

The statistical analysis of the lipid and metabolic profiles was performed in two steps. In the first step, analyses were performed using the procedure GLINMIX in SAS (version 9.2), where castration, histological type and the interaction between these variables were considered fixed effects in the statistical model. The following continuous probability distributions were tested for each variable: exponential, log-normal, gamma, Weibull, t-distribution, inverse Gaussian and normal. The criteria for obtaining the best fit for these distributions were the maximum likelihood and the relation between chi squared and degrees of freedom, which were considered better the closer they were to 1. For comparison between the maximum likelihood means, the *p*-value of each comparison was conclusive. All analyses were conducted using 0.05 as the critical level of probability for type I error.

Lipid and metabolic profiles evaluated during and after fish oil supplementation were analyzed using the GLINMIX procedure of SAS (version 9.2), where the use of fish oil was considered as a fixed effect in the statistical model. In this step, two orthogonal contrasts were performed: 1) Pre × 60: maximum likelihood means compared before and after 30 days of 60% omega-3 fish oil supplementation; and 2) Pre × 90: maximum likelihood averages compared before and after 30 days of 90% omega-3 fish oil supplementation. Survival curves were estimated using the Kaplan-Meier method and compared by the log-rank (Mantel-Cox) test and the Cox test in the univariate and multivariate analyses, respectively. In all cases, *p* < 0.05 was considered statistically significant. Survival analyses were performed using the software GraphPad Prism 5.0 (San Diego, CA, USA).

## Data Availability

The data that support the findings of this study are available from the corresponding author upon request.

## Abbreviations

AREG: Amphiregulin
BCS: Body condition score
CMT: Carcinoma in Mixed Tumors
DHA: Docosahexaenoic acid
EGFR: Epidermal growth factor receptor
EPA: Eicosapentaenoic acid
SEM: Standard error of the mean
HDL-C: *High-density lipoprotein* cholesterol
HUVEC: Human umbilical vein endothelial cell
IGF-1: Insulin-like growth factor 1
IL-1: Interleukin 1
IL-6: Interleukin 6
LDL-C: Low-density lipoprotein cholesterol
MC: Other carcinomas
MCF7: A breast cancer cell line – Michigan Cancer Foundation-7
MDA-MB-231: A breast cancer cell line
TG: Triglycerides
TNF-α: *Tumor necrosis factor* alpha
TNM: Tumor size (T), regional lymph node involvement (N), presence or absence of distant metastasis (M)
VLDL-C: Very low-density lipoprotein cholesterol
